# HDAC6-selective inhibitor CAY10603 ameliorates cigarette smoke-induced small airway remodeling by regulating epithelial barrier dysfunction and reversing

**DOI:** 10.1186/s12931-024-02688-3

**Published:** 2024-02-05

**Authors:** Qin Zhang, Liming Yan, Ye Lu, Xiaodong Liu, Yan Yin, Qiuyue Wang, Xiu Gu, Xiaoming Zhou

**Affiliations:** 1 National Center for Respiratory Medicine, Shenyang, China; 2 State Key Laboratory of Respiratory Health and Multimorbidity, Shenyang, China; 3 National Clinical Research Center for Respiratory Diseases, Shenyang, China; 4 Institute of Respiratory Medicine, Chinese Academy of Medical Sciences, Shenyang, China; 5 Department of Pulmonary and Critical Care Medicine, Center of Respiratory Medicine, China-Japan Friendship Hospital, Beijing, China; 6 Department of Pulmonary and Critical Care Medicine, Fourth Hospital of China Medical University, Shenyang, China; 7 Department of Respiratory and Critical Care Medicine, Shengjing Hospital of China Medical University, Shenyang, China; 8 Department of Respiratory and Critical Care Medicine, First Hospital of China Medical University, Shenyang, China; 9 Respiratory Department, Center for Pulmonary Vascular Diseases, Fuwai Hospital, National Center for Cardiovascular Diseases, Chinese Academy of Medical Sciences and Peking Union Medical College, Beijing, China

**Keywords:** HDAC6, COPD, Cigarette smoke, Airway remodelling, CAY10603

## Abstract

**Background:**

Small airway remodelling is a vital characteristic of chronic obstructive pulmonary disease (COPD), which is mainly caused by epithelial barrier dysfunction and epithelial-mesenchymal transition (EMT). Recent studies have indicated that histone deacetylase 6 (HDAC6) plays an important role in the dysregulation of epithelial function. In this study, we investigated the therapeutic effects and underlying mechanisms of an inhibitor with high selectivity for HDAC6 in COPD.

**Methods:**

Cigarette smoke (CS) exposure was used to establish a CS-induced COPD mouse model. CAY10603 at doses of 2.5 and 10 mg/kg was injected intraperitoneally on alternate days. The protective effects of CAY10603 against CS-induced emphysema, epithelial barrier function and small airway remodeling were evaluated using hematoxylin and eosin (H&E) staining, Masson’s trichrome staining, immunohistochemical staining, and western blot. The human lung bronchial epithelial cell line (HBE) was used to elucidate the underlying molecular mechanism of action of CAY10603.

**Results:**

HDAC6 levels in the lung homogenates of CS-exposed mice were higher than that those in control mice. Compared to the CS group, the mean linear intercept (MLI) of the CAY10603 treatment group decreased and the mean alveolar number (MAN)increased. Collagen deposition was reduced in groups treated with CAY10603. The expression of α-SMA was markedly upregulated in the CS group, which was reversed by CAY10603 treatment. Conversely, E-cadherin expression in the CS group was further downregulated, which was reversed by CAY10603 treatment. CAY10603 affects the tight junction protein expression of ZO-1 and occludin. ZO-1 and occludin expression were markedly downregulated in the CS group. After CAY10603treatment, the protein expression level of ZO-1 and occludin increased significantly. In HBE cells, Cigarette smoke extract (CSE) increased HDAC6 levels. CAY10603 significantly attenuated the release of TGF-β1 induced by CSE. CAY10603 significantly increased the E-cadherin levels in TGF-β1 treated HBE cells, while concurrently attenuated α-SMA expression. This effect was achieved through the suppression of Smad2 and Smad3 phosphorylation. CAY10603 also inhibited TGF-β1 induced cell migration.

**Conclusions:**

These findings suggested that CAY10603 inhibited CS induced small airway remodelling by regulating epithelial barrier dysfunction and reversing EMT via the TGF-β1/Smad2/3 signalling pathway.

**Supplementary Information:**

The online version contains supplementary material available at 10.1186/s12931-024-02688-3.

## Background

Chronic obstructive pulmonary disease (COPD) is a chronic inflammatory airway disease mainly characterised as a chronic airway limitation caused by noxious particles and gases [1, 2]. The small airways are the major sites of obstruction in patients with COPD [3]. The number of patent terminal and transitional bronchioles is reduced in patients with COPD, but the damage can accumulate without being noticed [1, 4]. The remaining small airways are thickened and become more obstructed with disease progression as small airway remodelling [5–7], while emphysema is reported to be a later variable secondary phenomenon [5, 8]. Epithelial barrier dysfunction in COPD underlies the impaired repair response of the injured epithelium and its inability to redifferentiate into a functionally intact epithelium, thus leading to small airway obstruction [9–11]. Epithelial-mesenchymal transition (EMT) is a pathophysiological process observed in COPD wherein epithelial cells lose their polarity and transform into mesenchymal cells [10]. EMT promotes the progression of small airway remodelling and fibrosis in patients with COPD. Therefore, exploring therapies targeting epithelial barrier dysfunction and small airway remodelling in COPD may be beneficial for COPD management.

Genome wide studies of lung tissue from patients with COPD have revealed that epigenetic changes contribute to individual susceptibility to COPD, representing one of key mechanism of COPD progression [1, 12]. The activity of histone deacetylase (HDAC), as a key molecule in many inflammatory processes [13, 14], is reported to be reduced in the lungs of affected patients in proportion to the severity of airflow limitation [15]. However, the mRNA expression of HDAC2, HDAC5, and HDAC8 and the protein expression of HDAC2 are decreased in lung tissues with increasing disease severity [15]. Notably, 18 HDACs have been identified in mammals, and specific HDACs appear to be differentially regulated by different groups of genes [16]. HDAC6, different from other HDAC isoenzymes, is ubiquitously expressed and predominantly located in the cytoplasm, where it mediates deacetylation and regulates microtubule-dependent cell motility [17]. Inhibition of HDAC6 has been shown to attenuate the disruption of lung endothelial barrier integrity induced by cigarette smoke (CS) [18]. Another study [19] indicated that HDAC6 inhibitors protect against CS-induced mucociliary clearance disruption. However, the effects of HDAC6 inhibitors on CS-induced EMT and small airway remodelling remain unclear.

In this study, we hypothesised that HDAC6 inhibitors play a protective role against CS-induced EMT and epithelial barrier dysfunction in the small airway, thereby thus inhibiting small airway remodeling. CAY10603, a small molecule inhibitor that is highly potent for HDAC6 and has a good selective profile, was used in this study. The protective effects of CAY10603 against CS-induced EMT and small airway remodelling were evaluated using a CS-exposed mouse model and in vitro experiments.

## Materials and methods

### Chemicals and reagents

For in vivo and in vitro experiments, the highly selective HDAC6 inhibitor, CAY10603 (molecular weight: 446.5), was purchased from Selleck Chemicals (Shanghai, China) with purity > 99.04%.

### Animals

Male C57BL/6J mice 8–10 weeks of age were purchased from Liaoning Changsheng Biotechnology Company (Benxi, China). The mice were housed and fed at the First Hospital of China Medical University, the Institute of Respiratory Disease, under quiet and controlled specific pathogen-free conditions with a temperature of between 21 and 22 °C and humidity between 50% and 60% under a 12-h/12-h light/dark cycle. Forty-eight mice (weight range between 18 and 20 g) were randomly selected and divided into four groups (*n* = 12 per group): (I) control (CON); (II) CS exposed (CS); (III) CS + 2.5 mg/kg CAY10603 (CS + L-CAY); (IV) CS + 10 mg/kg CAY10603 (CS + H-CAY). Mice were passively exposed to CS for 12 consecutive weeks (20 cigarettes/exposure session, 60 min per session, twice/day, 6 days/week) or room air (AIR), beginning at 8 weeks of age, using –the HOPE-MED8050 inhalation exposure system (HOPE Company, Tianjin, China), a whole-body smoke exposure system, as previously described [20–22]. Non-filtered Marlboro cigarettes (Philip Morris Companiy, 0.8 mg of nicotine, 10 mg of Tar, and 10 mg of carbon monoxide per cigarette) were used for the CS. The total particulate matter concentrations in the exposure chamber were between 150 and 180 mg/m^3^. Mice in the control group were exposed to room air for 12 weeks. In the groups receiving CAY10603 treatment, mice were intraperitoneally injected with CAY10603 on alternate days. CAY10603 was dissolved in DMSO, and the dose of CAY10603 was determined from the literature [23]. The mice were euthanised at the end of 12 weeks, 24 h after the last exposure to CS, and the lungs were harvested for subsequent experiments.

The experimental protocol was approved by the Ethics Committee of China Medical University, and Shengjing Hospital of China Medical University, and all animal care and procedures were performed according to the recommendations of the Guide for the Care and Use of Laboratory Animals (IACUC Issue No. KT2018061 and 2019PS369K).

### Lung tissue and sample preparation

In each group, the left lungs of 6 mice were infused with 0.3 mL PBS for three times to harvest harvest of bronchoalveolar lavage fluid (BALF), while the left lung tissues of the other 6 mice were ligated, removed, and immersed in 10% neutral formaldehyde for 7-day fixation, followed by paraffin embedding using standard procedures. The paraffin sections (4 μm) were prepared and used for subsequent histopathological studies. The left lungs of some mice were perfused with 0.3mL of ice-cold PBS/time × 3 times, and BAL fluid was harvested. To obtain BAL fluid, the trachea was exposed using scissors and the left main bronchus was ligated. A 23G needle was used to inject 0.3 mL of cold PBS containing 0.1 mM EDTA into the right lung, followed by the retrieval of BALF from the lungs. The right lung tissues were removed and stored at -80 ℃ until required for analysis.

### Tissue histology

Tissue Sect. (4 μm) were stained with haematoxylin and eosin (H&E), Masson’s trichrome and periodic acid–Schiff (PAS) staining to examine thehistological changes. The morphology of emphysema changes was compared by measuring the mean linear intercept (MLI) and mean alveolar number (MAN), as previously described (×100 magnification), with a smaller value indicating more severe emphysema [24].

### Airway remodelling assay

All slides were examined using light microscopy at ×400 magnification to assess airway remodelling. Bronchioles with a 150–200 μm internal diameter were selected in a blinded manner before observing and photographing. The perimeter of the bronchial basement membrane (Pbm) was measured as a calculation reference for the airway [25]. The airway epithelial (µm^2^) and collagen deposition area (µm^2^) were assessed in a minimum of four small airways (basement membrane perimeter < 1000 μm) per section according to the previously described method [22, 26]. Peribronchial collagen deposition was examined by Masson’s trichrome staining, and goblet cell hyperplasia was examined using PAS staining [22, 25]. Data were quantified using ImageJ software (version 1.50; National Institutes of Health, Bethesda, MD, USA) and normalised to the basement membrane perimeter (µm) [22, 25, 26].

### Airway inflammation evaluation

We employed an inflammation score based on H&E staining and the level of inflammatory cytokines in the BALF to evaluate the airway inflammation status. The inflammation score was determined based on the degree of peribronchial and perivascular inflammation scored on a subjective scale of 0 (no) to 4 (severe) in a blinded manner by three examiners, as previously described [27]. Scoring was performed by comparing standardised figures presenting the grades. The inflammation score was defined as the sum of the peribronchial and perivascular scores (0–8). Assessment of the level of pro-inflammatory cytokines TNF-α and IL-6 level was conducted in BALF supernatant samples and quantified using commercially available ELISA kits (R&D System, Minneapolis, Canada).

### Immunohistochemistry

Lungs were perfused, inflated, formalin-fixed, paraffin-embedded and sectioned (4–6 μm). Longitudinal sections of the left lung were rehydrated,deparaffinised, and stained with antibodies against ZO-1, occludin, Muc5ac, α-SMA, and E-cadherin (1:200 dilution, Abcam), followed by incubation with HPR-linked secondary antibody (1:1,000 dilution, Abcam) for 30 min at room temperature. DAB (Maixin Technology Co., Ltd. Fuzhou, China) solution was used for the chromogenic reactions. Sections were observed under a microscope. Quantification of the immunohistochemistry positive staining area was assessed with ImageJ software and normalised to the basement membrane perimeter (µm) [22, 25, 26].

### Western blot analysis

Western blot analysis was performed as previously described [28]. The primary antibodies were as follows: HDAC6 rabbit monoclonal antibody (1:1000; Cell Signaling Technology, 7612), ZO-1 rabbit monoclonal antibody (1:1000; Cell Signaling Technology, 13,663), Occludin rabbit monoclonal antibody (1:1000; Cell Signaling Technology, 91,131), E-cadherin mouse monoclonal antibody (1:1000; Cell Signaling Technology, 14,472), α-smooth muscle actin rabbit monoclonal antibody (1:1000; Cell Signaling Technology, 19,245), Acetyl-α-tubulin Antibody (1:1000; Cell Signaling Technology, 3971), α-tubulin rabbit monoclonal antibody (1:1000; Cell Signaling Technology, 2125), SMAD2 rabbit polyclonal antibody (1:1000; Proteintech, 12570-1-AP), Phospho-SMAD2 rabbit polyclonal antibody (1:1000; Cell Signaling Technology, 18,338), SMAD3 mouse monoclonal antibody (1:1000; Proteintech, 66516-1-Ig), Phospho-SMAD3 rabbit polyclonal antibody (1:1000; Cell Signaling Technology, 9520), β-Actin mouse monoclonal antibody (1:1000; Santa Cruz Biotechnology, sc-8432).

### Cigarette smoke extract (CSE) preparation

CSE was prepared based on a previous report [28]. Briefly, one cigarette (Marlboro, Longyan Tobacco Industrial Co. Ltd., Fujian, China; tar: 10 mg/cigarette; nicotine: 0.8 mg/cigarette; carbon monoxide: 11 mg/cigarette) was lit and the smoke was slowly pumped into a 10mL medium for a total of 5 min. The pH of the solution was adjusted to 7.4. After filtering the CSE (0.22 μm; Merck Millipore, SLGS033SS) twice to remove insoluble particles, the resulting solution was defined as having a 100% CSE concentration. Subsequently, the 100% CSE was diluted to the required concentration of the working solution with the medium. The CSE working solution was considered effective within 1 h.

### Cell culture

The human lung bronchial epithelial cell line (HBE) was purchased from the China Infrastructure of Cell Line Resources and cultured in PRMI 1640 medium containing 10% fetal bovine serum (FBS) at 37 °C in a 5% CO_2_ atmosphere. In the TGF-β1 treatment experiments, the cells were Subsequently, t with 5 µg/mL TGF-β1 (Biosource, Camarillo, CA, USA) for TGF-β1 treatment experiments or 6% CSE for CSE treatment experiments, either in the presence of CAY10603 at the indicated concentrations and time points. After centrifugation, the cells and supernatant were collected for subsequent experiments. The release of TNF-α and IL-6 was assessed using commercially available ELISA kits (FANKEW, Shanghai, China).

### Transwell assay

The cell migration ability was measured using the Transwell migration assay as previously described [29]. Briefly, HBE cells were cultured in a serum-free medium in the upper chamber, while a growth medium containing FBS was added to the bottom chamber as a chemical attractant. Cells were incubated with 5% CO_2_ at 37 °C; 24 h later, the cells were fixed and then stained with 0.1% crystal violet. The number of migrating cells was quantified under a microscope.

### Statistical analysis

The results are depicted as the mean ± SEM. Statistical analysis was performed using a one-way analysis of variance (ANOVA) test, followed by a Newman-Keuls comparison using GraphPad Prism software (San Diego, CA, US). *p* < 0.05 was considered significant.

## Results

### CAY10603 alleviated CS-induced pulmonary emphysema in mice

**Fig. 1:**
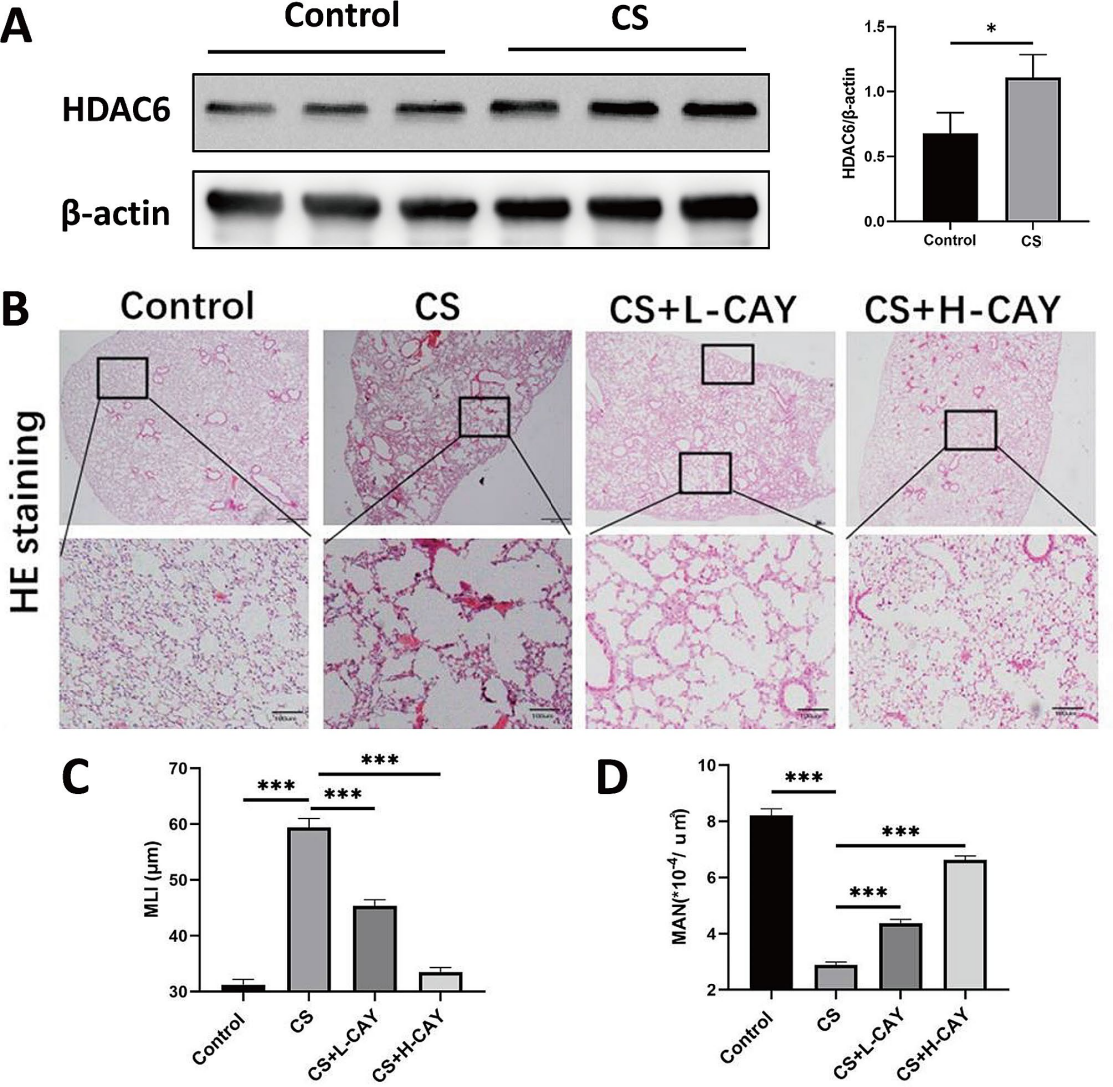
CAY10603 alleviated CS-induced pulmonary emphysema in mice model. **(A)**: Western blotting analysis of HDAC6 protein expression in the lung tissue from mice from different groups (*n* = 3 mice/group). (**B**): Micrographs of mice lungs stained with H&E (×100): (1) control group, (2) CS exposed group (CS), (3) CS + L-CAY group with 2.5 mg/kg CAY10603, (4) CS + H-CAY group with 10 mg/kg CAY10603; (**B**): Mean linear intercepts (MLI) in each group; (**C**): Mean alveolar number (MAN) in each group (*n* = 6 mice/group). Bars represent mean ± SEM values. *p < 0.05, **p < 0.01, ***p < 0.001

The HDAC6 levels in the lung homogenates of mice in CS group were higher than those in the control group **(**Fig. 1A**)**. Lung sections from the mice in the CS group showed alveolar enlargement and alveolar septum rupture, which are typical features of pulmonary emphysema. Similar pathological features were observed in the CS + 2.5 mg/kg CAY10603 group. Conversely, lung sections of the CS + 10 mg/kg CAY10603 group showed characteristics analogous to those of the control group, in which the alveoli arrangement was regular and the alveolar septa was thin (Fig. 1B). The MLI in the CS group was significantly from the mice in the CS group, the MLI in the CAY10603 treatment group was lower than that in the CS group. Moreover, the MLI in CS + 10 mg/kg CAY10603 group was similar to that in the control group (Fig. 1C). The MAN measurements exhibited the opposite trend, in that the MAN of the CS group was significantly lower than that of the control group, while CAY10603 treatment increased the MAN, indicating that CAY10603 improved emphysema in the lungs of CS-exposed mice (Fig. 1D).

### Effect of CAY10603 on epithelial barrier dysfunction

**Fig. 2:**
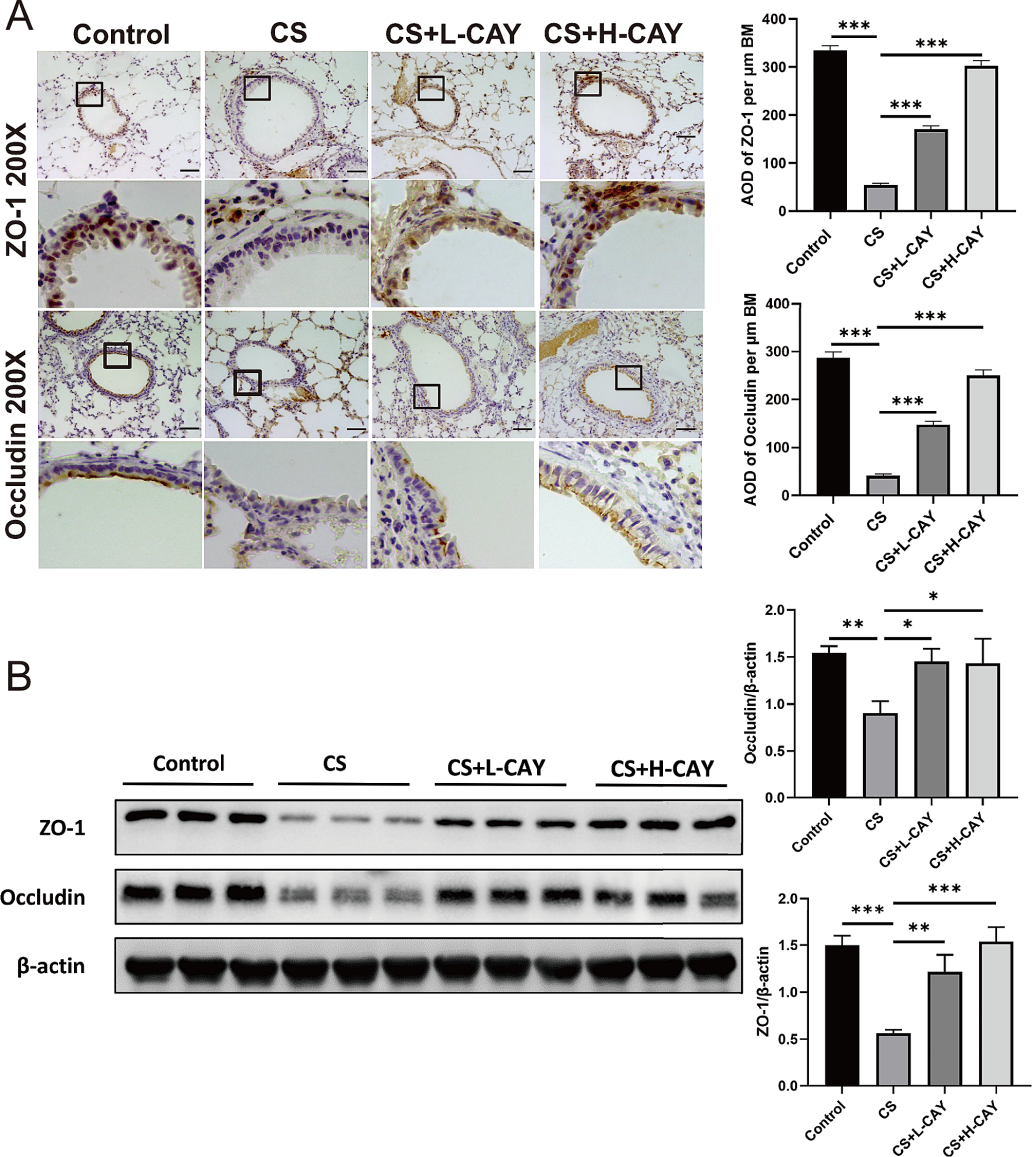
CAY10603 affected CS-induced epithelial barrier dysfunction in mice model. (**A**): The IHC staining was performed to ZO-1 and Occludin protein expression (brown) in the sections of lung tissue (scale bar: 50 μm ): (1) control group, (2) CS exposed group (CS), (3) CS + L-CAY group with 2.5 mg/kg CAY10603, (4) CS + H-CAY group with 10 mg/kg CAY10603; The amount of expression was quantified by AOD (*n* = 6 mice/group); (**B**): Western blot was performed to ZO-1 and Occludin protein expression in the sections of lung tissue (*n* = 3 mice/group). The expression levels of related proteins were expressed by relative fold change. All data were shown as means ± SEM. *p < 0.05, **p < 0.01, ***p < 0.001

The expression levels of the barrier function-related protein, ZO-1 and occludin, were detected by IHC and Western blot. Compared with the control group, the expressions of ZO-1 and occludin was markedly downregulated in the CS group, both of which increased significantly after CAY10603 treatment (Fig. 2A). Western blot results revealed similar results (Fig. 2B). The above results indicate that CAY10603 affects epithelial barrier function by affecting the tight junction protein expression of ZO-1 and occludin.

### CAY10603 attenuates airway inflammation, airway mucus hypersecretion, and airway remodelling in the CS mouse model

**Fig. 3:**
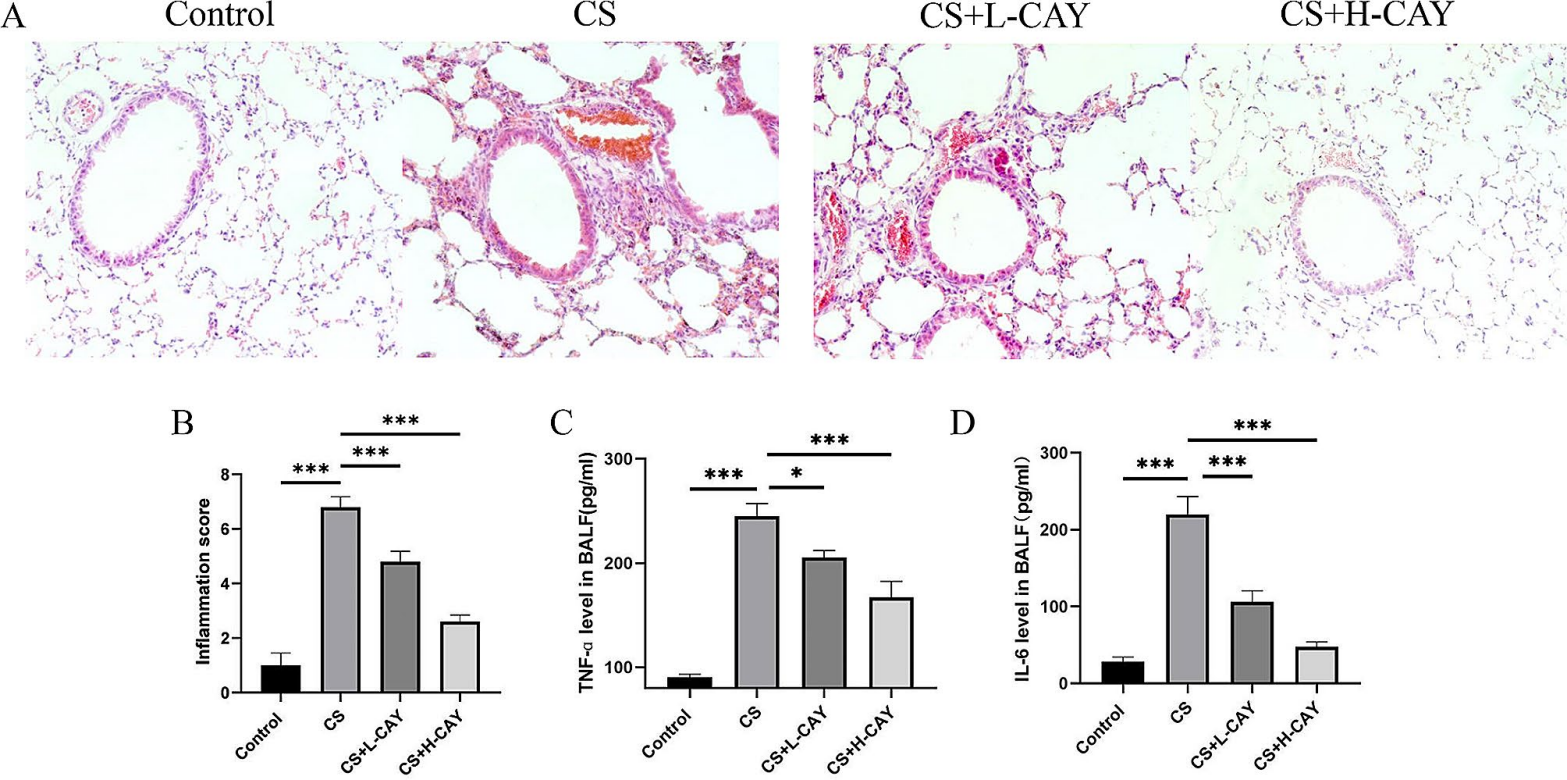
CAY10603 reduced CS-induced airway inflammation in mice model. (**A**): Micrographs of mice lung tissue sections of stained with H&E; (**B**): Inflammation scores of the HE-staining results; (**C**): Effects of CAY10603 on TNF-ɑ in BALF; (**D**): Effects of CAY10603 on IL-6 in BALF. The comparison was between two groups, (I) control group (CON); (II) CS exposed group (CS); (III) CS + L-CAY group with 2.5 mg/kg CAY10603; (IV) CS + H-CAY group with 10 mg/kg CAY10603. (*n* = 6 mice/group) *p < 0.05, **p < 0.01, ***p < 0.001. Bars represent mean ± SEM values

CS-induced oxidative stress produces inflammatory factors, such as IL-6 and TNF-α, which amplify the inflammatory process and induce changes in airway structure. The measurement of inflammation scores revealed that, compared to the control group, inflammatory cell infiltration around the peribronchia was observed in the CS group but was significantly reduced in the CAY10603 treatment groups (Fig. 3A–B). Next, the expression of IL-6 and TNF-α in BALF after 12 weeks of CS exposure, was measured. The results revealed that the TNF-α levels in the BALF of CS mice were significantly increased compared to those in the control group and were reversed by CAY10603 treatment (Fig. 3C). The concentrations of IL-6 showed similar results (Fig. 3D). The results demonstrated that CAY10603 reduced airway inflammation.

**Fig. 4:**
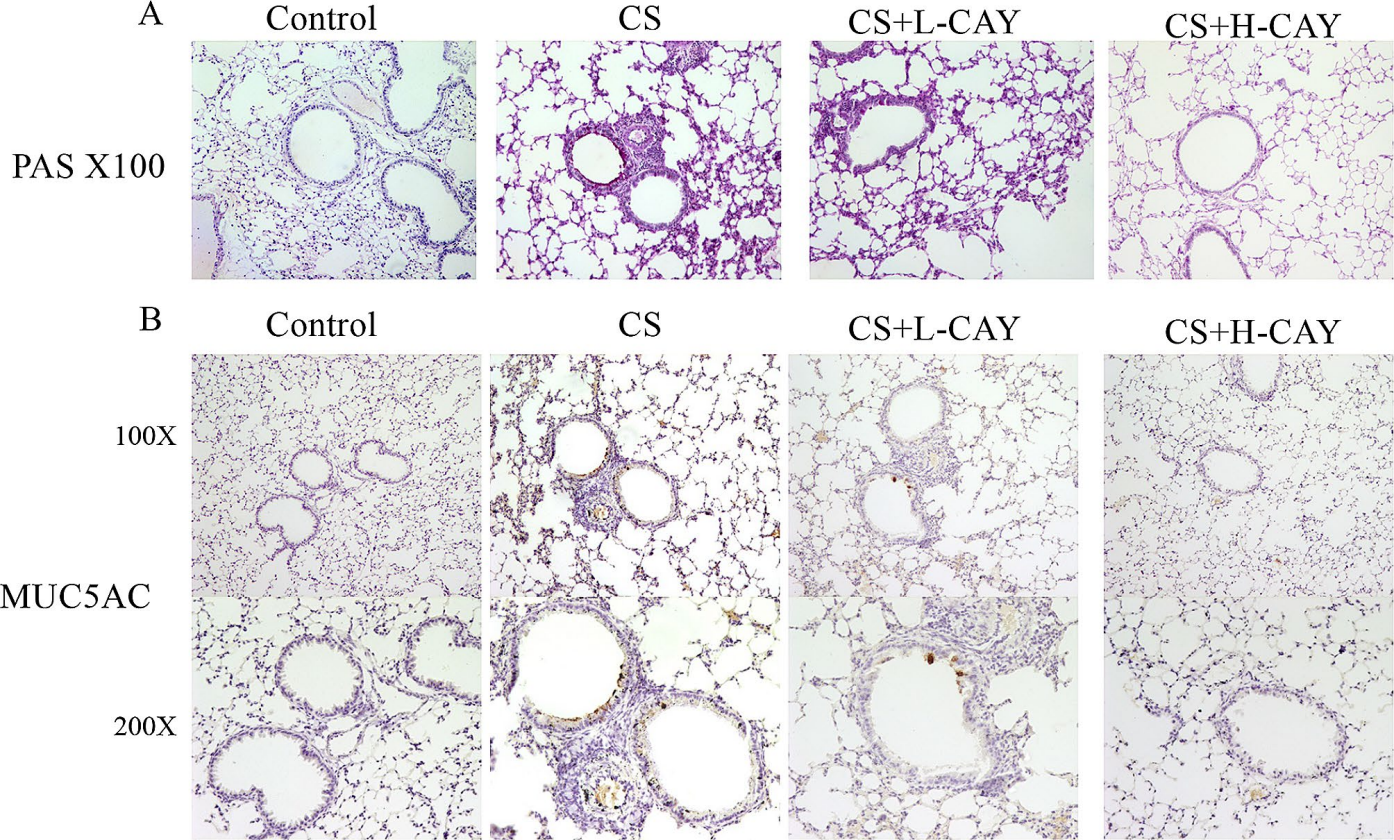
CAY10603 reduced CS-induced airway mucus hypersecretion in mice model. (**A**): PAS-staining of lung tissue sections of mice were shown at 100× magnification. (1) control group, (2) CS exposed group (CS), (3) CS + L-CAY group with 2.5 mg/kg CAY10603, (4) CS + H-CAY group with 10 mg/kg CAY10603; (**B**): The IHC staining was performed to assess the expression of Muc5ac protein (brown) in the lung tissue sections of mice

PAS staining microscopy images were shown in Fig. 4A. The results showed that goblet cell hyperplasia increased in the CS group and was subsequently suppressed in the CAY10603 treatment groups. Similarly, the expression of the major airway mucus protein Muc5ac was elevated following CS exposure; however, this upregulation was mitigated by CAY10603 treatment (Fig. 4B). The results demonstrated that CAY10603 effectively reduced airway mucus hypersecretion.

**Fig. 5:**
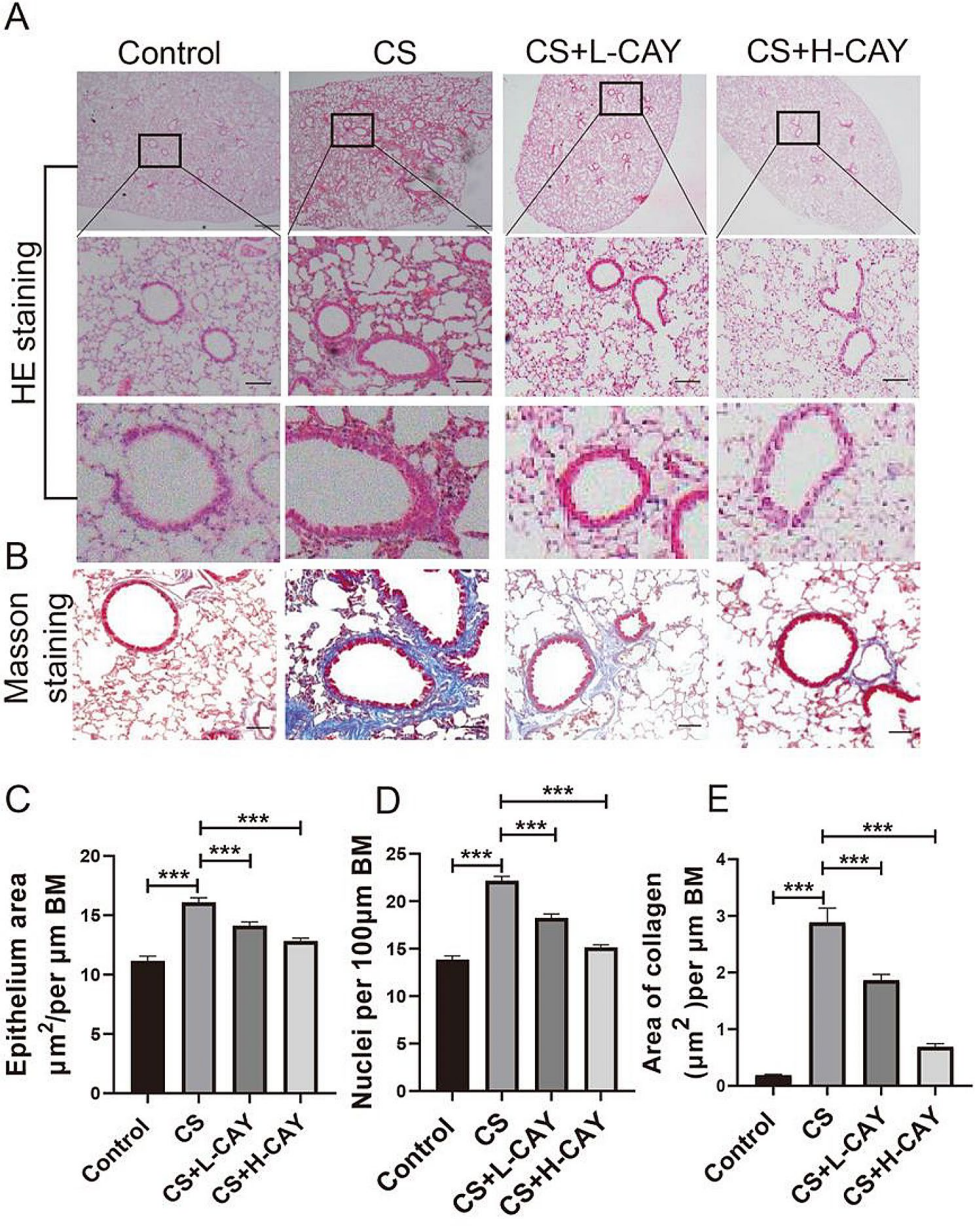
CAY10603 reduced CS-induced small airway remodeling in mice model. (**A**): H&E staining of small airway (scale bar: 50 μm ): (1) control group, (2) CS exposed group (CS), (3) CS + L-CAY group with 2.5 mg/kg CAY10603, (4) CS + H-CAY group with 10 mg/kg CAY10603; (**B**): Massion-staining of small airway; (**C**): The epithelium area by H&E staining was assessed in small airway and standardized by airway basement membrane (µm); (**D**): The cell (nuclei) number of the epithelium by H&E staining was assessed in small airway and standardized by airway basement membrane (µm); (**E**): The peribronchiolar collagen deposition rate in each groups, standardized by airway basement membrane(µm). Bars represent mean ± SEM values (*n* = 6 mice/group). *p < 0.05, **p < 0.01, ***p < 0.001

Associated changes in the small airways occur in patients with COPD. Representative H&E and Masson stained microscopic images were shown in Fig. 5A–B. Lung sections from mice in the CS group showed small airway wall thickening and collagen accumulation outside the airways, while similar pathological features were observed in the CS + 2.5 mg/kg CAY10603 group. Conversely, lung sections of the CS + 10 mg/kg CAY10603 group were analogous to those of the control group, in which the small airway wall was approximately a monolayer. Compared to the control group, the epithelial area, nuclei, and collagen area of CS group were significantly increased **(**Fig. 5C-E**)**. The epithelium area, nuclei, and collagen areas of CAY10603 group were lower than those of the CS group, indicating that CAY10603 could reduce stinginess, wall thickening, and collagen deposition.

### CAY10603 attenuated CS-induced EMT in mice

**Fig. 6:**
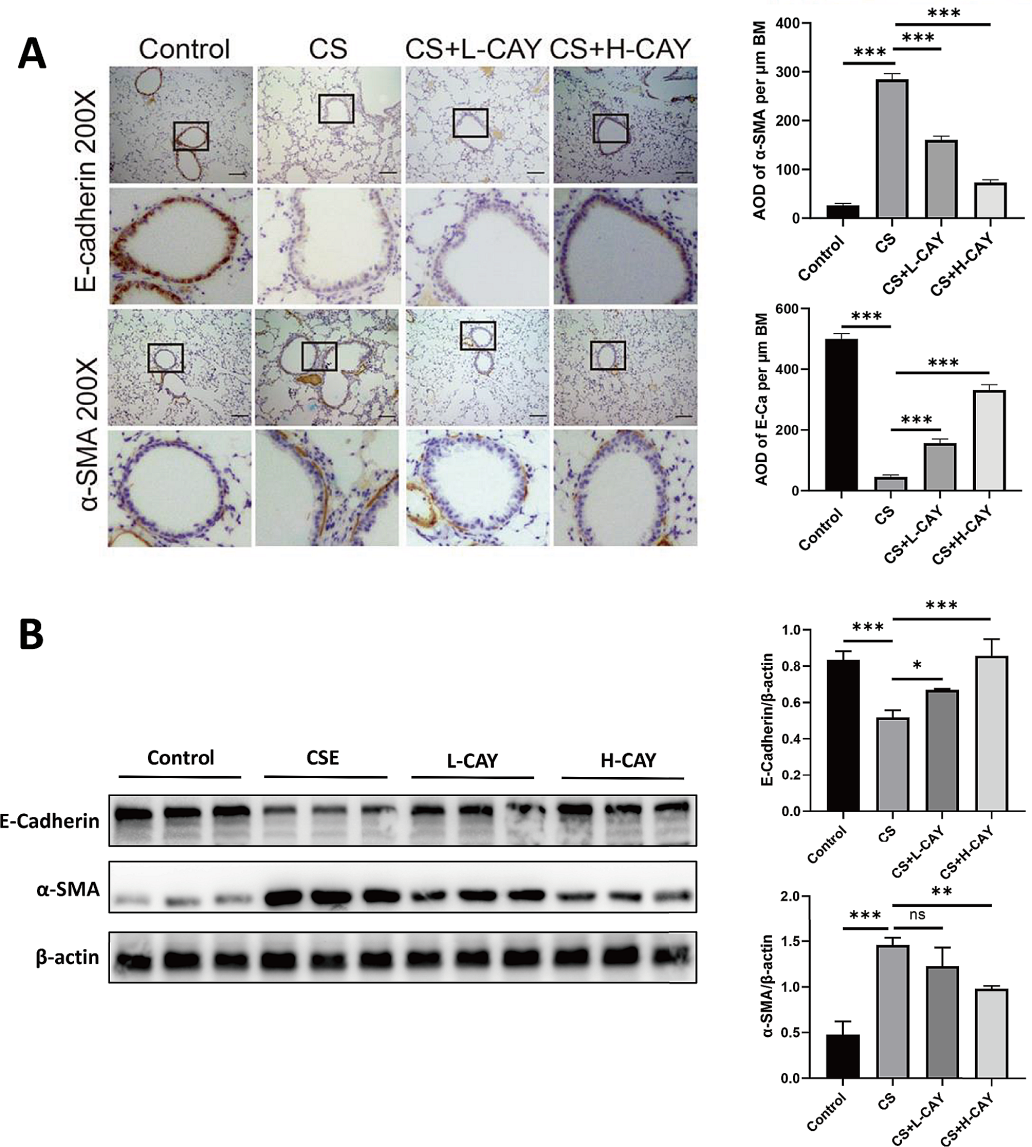
CAY10603 attenuated CS-induced EMT in mice model. (**A**): The IHC staining was performed to E-cadherin and α-SMA protein expression (brown) in the sections of lung tissue (scale bar: 50 μm ): (1) control group, (2) CS exposed group (CS), (3) CS + L-CAY group with 2.5 mg/kg CAY10603, (4) CS + H-CAY group with 10 mg/kg CAY10603; The amount of expression was quantified by AOD (*n* = 6 mice/group). (**B**): The protein expression of α-SMA, and E-cadherin in mice lung tissue was shown by Western blot. The expression levels of related proteins were expressed by relative fold change (*n* = 3 mice/group). All data were shown as means ± SEM. *p < 0.05, **p < 0.01, ***p < 0.001

The expression levels of EMT-related protein molecules, such as E-cadherin and α-SMA, were detected by IHC and western blot. Compared to the the control group, the expression of α-SMA was markedly upregulated in the CS group and reversed by CAY10603 treatment. Conversely, E-cadherin expression was downregulated. Upregulated expression was observed in the groups treated with CAY10603 **(**Fig. 6A**)**. The western blot results showed similar results **(**Fig. 6B**)**. These changes in EMT marker expression suggested that inhibition of HDAC6 could reverse the airway EMT process and HDAC6 might play an important role in airway remodelling and EMT.

**Fig. 7:**
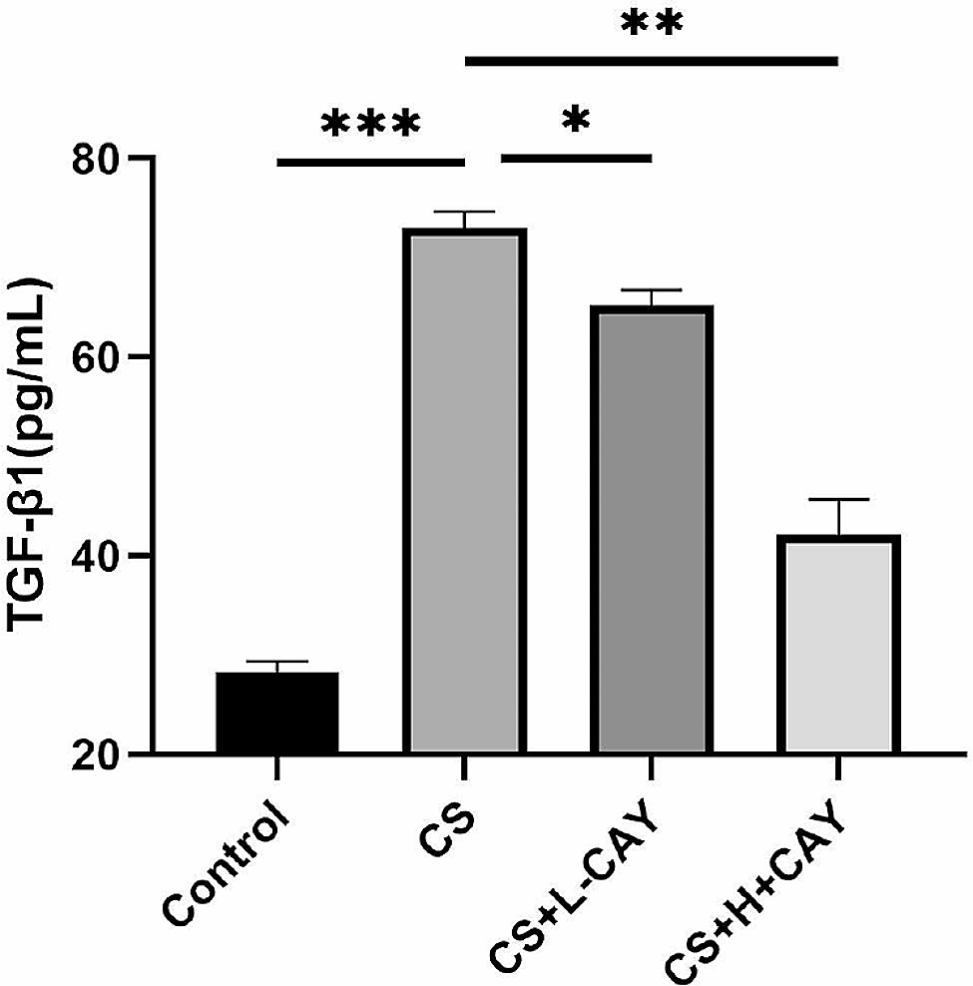
Effects of CAY10603 on TGF-β1 in BALF. The level of TGF-β1 in the mice BALF was detected by ELISA assay: (I) control group; (II) CS exposed group (CS); (III) CS + L-CAY group with 2.5 mg/kg CAY10603; (IV) CS + H-CAY group with 10 mg/kg CAY10603. Bars represent mean ± SEM values (*n* = 6 mice/group). *p < 0.05, **p < 0.01, ***p < 0.001

The expression of TGF-β1, which plays an important role in EMT, was detected in BALF. The ELISA results showed that compared to control mice, the content of TGF-β1 in the BALF of mice in the CS group and CS + 2.5 mg/kg CAY10603 group was significantly increased. The content of TGF-β1 in the CS + 10 mg/kg CAY10603 group was significantly decreased, indicating that CS increased the level of TGF-β1 in the lungs, while CAY10603 inhibited this change (Fig. 7).

### CAY10603 suppressed the release of inflammatory factors, cell migration, and the TGF-β1-induced EMT process in HBE cells

**Fig. 8:**
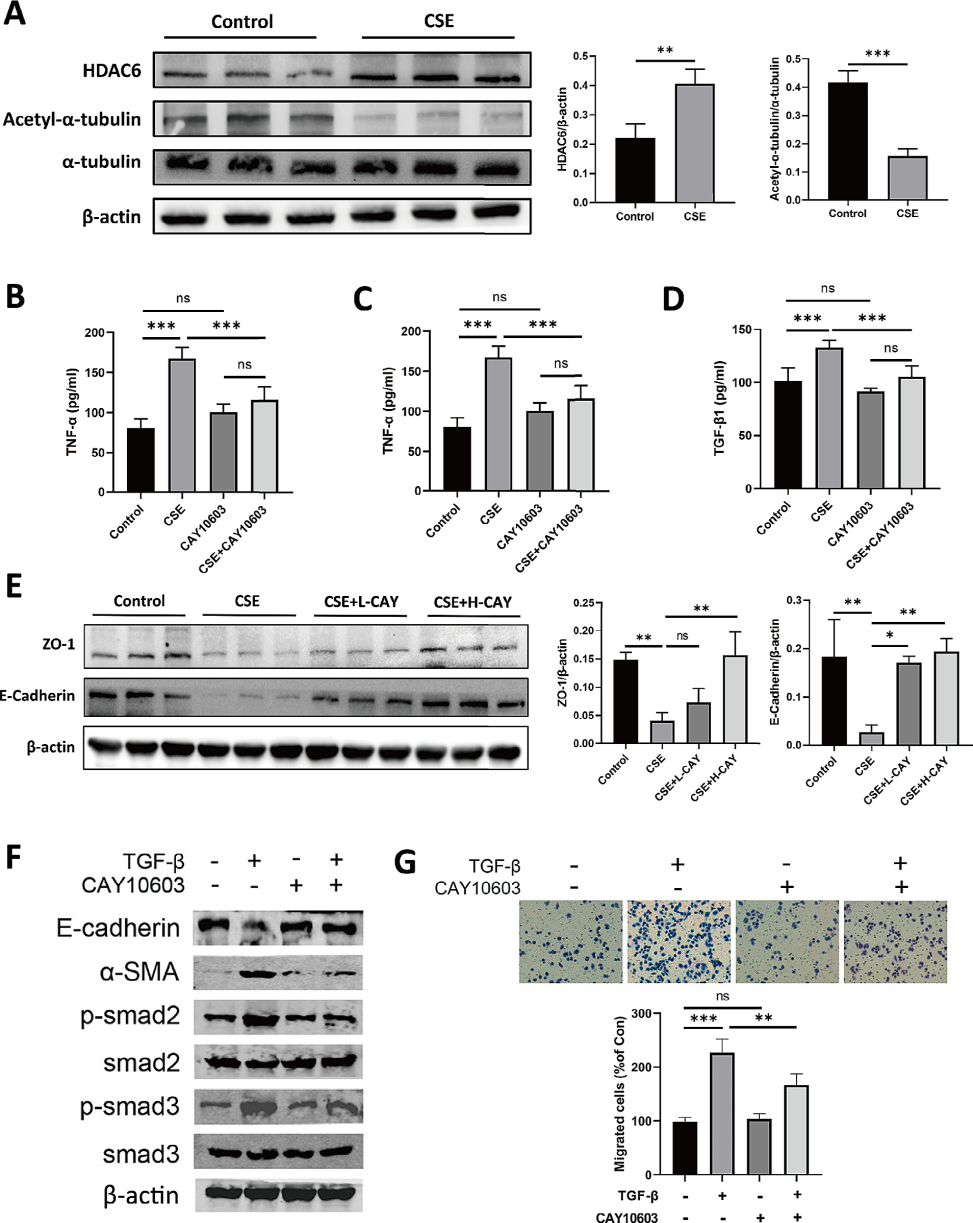
Effect of CAY10603 on TGFβ1 induced EMT in vitro. (**A**): Western blotting analysis of HDAC6, Acetyl-α-tubulin and α-tubulin level in HBE cells treated with CSE or PBS (*n* = 3). (**B-D**): The level of TNF-ɑ (**B**), IL-6 (**C**) and TGF-β1 (**D**) released from HBE cells treated with CSE or/and CAY10603 was detected by ELISA assay (*n* = 5). (**E**) Western blotting analysis of ZO-1 and E-Cadherin level in HBE cells of different groups (*n* = 3): (I) control group; (II) CSE treatment group (CSE); (III) CS + L-CAY group with 25 nMol CAY10603; (IV) CS + H-CAY group with 50 nMol CAY10603. (**F**): Protein expression of E-cadherin, α-SMA, p-smad2, smad2, p-smad3, smad3 was shown by Western blot (*n* = 3). (**G**): The transwell migration assay examined TGFβ1-induced cell migration in HBE cells with CAY10603 (*n* = 3). The number of cells was shown by crystal violet staining. Bars represent mean ± SEM values. *p < 0.05, **p < 0.01, ***p < 0.001

First, we investigated CSE-induced HDAC6 expression in HBE cells. The results showed that the HDAC6 levels in the CSE-treated cells were significantly higher than those in the control group (Fig. 8A). Correspondingly, we observed a decrease in the acetyl-α-tubulin level (Fig. 8A), which is widely recognised to be the first substrate of HDAC6 [30].

CAY10603 inhibited the CSE-induced release of inflammatory factors in HBE cells. The ELISA result showed that the TNF-α and IL-6 levels in the CSE-treated group were significantly increased compared to those in the control group and were reversed by CAY10603 (Fig. 8C–D).

EMT contributes to airway remodeling and is considered a critical mechanism in the pathogenesis of COPD [9, 31]. It is known that disruption of airway epithelial barrier triggers EMT [32, 33], in which TGF-β1 is a key inducer [34]. The result showed that CAY10603 attenuated TGF-β1 level in CSE-treated HBE cells (Fig. 8D) and protected against CSE-induced decrease in the epithelial barrier function-related protein level (ZO-1 and E-Cadherin) (Fig. 8E). We next investigated the effect of CAY10603 on EMT in airway epithelial cells. Compared to the control group, TGF-β1 induced the EMT process in HBE cells, which showed downregulation of E-cadherin expression and upregulation of α-SMA expression level; meanwhile, the phosphorylation levels of smad2 and smad3 were significantly upregulated by TGF-β1 (Fig. 8F). Importantly, CAY10603 could rescue the TGF-β1 induced EMT process and upstream regulatory pathway. Extensive studies have shown that TGF-β1-induced EMT processes enhance the migratory capacity of airway epithelial cells. Therefore, we next investigated the effects of CAY10603 on the migratory capacity of HBE cells. As expected, CAY10603 significantly inhibited the TGF-β1-induced enhancement of HBE cell migratory capacity (Fig. 8G). In conclusion, these results suggest that CAY10603 inhibits the TGF-β1-induced EMT process and cell migration.

## Discussion

In the present study, we demonstrated that CAY10603 improved emphysema and airway inflammation induced by CS, both in vivo and in vitro. The mechanism of action of HDAC6 may be associated with regulating epithelial barrier dysfunction and reversing EMT via the TGF-β1/Smad2/3 signaling pathway.

EMT is the process by which epithelial cells lose polarity and transform into mesenchymal cells under specific conditions [35]. Recent studies [36] have suggested that airway remodelling in COPD is mainly related to type 2 EMT, which involves tissue fibrosis. Similar to EMT, endothelial to mesenchymal transition (EndMT) has been reported to be involved in vascular remodeling in COPD [37]. The mechanism of EMT has been extensively studied in the pathogenesis of tumour cell infiltration and metastasis. Moreover recent studies have found that EMT occurs in the small airway epithelium of patients with COPD [38], and may play an important role in the occurrence and development of airway remodelling. Smoking is one of the most important causes of COPD [39], and current studies have mainly focused on the effects of CS on the EMT in COPD. CS acts on EMT through various pathways, including oxidative stress, destruction of cell connections, and destruction of the cytoskeletal structure. Apoptosis mediated by reactive oxygen species (ROS) can also act on EMT through the PI3K/AKT/NFKB/MMP-9 signalling pathway in cancer [40]. In COPD, ROS could promote epithelial phenotypic transformation, resulting in abnormal proliferation and differentiation of epithelial cells, leading to subepithelial collagen deposition [41]. Recent studies have observed that CS can lead to EMT in both alveolar and airway epithelium. CS has also been shown to promote EMT in alveolar epithelial cells through the WNT/β-catenin signaling pathway, resulting in impaired alveolar repair ability [42]. As COPD begins with small airway dysfunction our study focused on changes in the small airways and airway epithelium in COPD. The mechanism by which CS promoting EMT in airway epithelial cells is also under study, which may involve the TGF-β/Samd [43], WNT/β-catenin, Hedgehog (Hh) [44], urokinase plasminogen activator receptor (uPAR) [45], and Notch signaling pathways [46]. Among them, the TGF-β/Samd pathway has been relatively studied. TGF-β mainly phosphorylates the Smad complex, which can translocate into the nucleus and promote the expression of EMT transcription-induced genes [11]. In our study, similar manifestations were found, including increased TGF-β in BALF after CS exposure and increasedpSmad2/3 in cell experiments.

Multiple previous studies have demonstrated that HDAC6 inhibitors effectively suppress TGF-β1/Smad pathway activation [47–49]. However, the specific mechanism by which HDAC6 facilitates the activation of Smad3 remains unclear. One potential mechanism involves the regulation of Smad7 expression. In a study conducted by Chen et al. [49], Acy-1215, a specific inhibitor of HDAC6, significantly suppressed the increased expression of TGF-β1 and p-Smad3 induced by unilateral ureteral obstruction and partially restored the expression of Smad7. Smad7 acts as a negative regulatory factor in the TGF-β1/Smad3 pathway, where its upregulation can attenuate the recruitment of Smad3 to phosphorylated TGF-β1 receptors, resulting in the downregulation of Smad3 phosphorylation [50]. Therefore, the upregulation of Smad7 induced by HDAC6 may attenuate the effects of the TGF-β1/Smad3 signalling pathway. However, further investigation is required to test this hypothesis.

The barrier function of the airway epithelium, a structure of interconnected cells that form the first barrier against environmental damage e and is maintained by tight junctions (TJs) and adherens junctions (AJs), limits the permeability to inhaled pathogens and environmental stressors [51]. TJs, the apical portion of the cell surface, are composed of the transmembrane proteins claudin (CLDN), occludin (OCLN), and junction adhesion molecules (JAMs) [52]. In addition, zonula occludens (ZO)-1, ZO-2, ZO-3, Par-3, and Par-6 are also associated with TJ formation [53]. TGF-β1 has been shown to prevent CSE-induced tight junction disruption and barrier function loss. TGF-β1 treatment of CSE human bronchial epithelial cells (16HBE14o^−^) has been shown to restore ZO-1 and ZO-2 protein levels [54]. Other cell experiments [55] have shown that HDAC6 deacetylates the promoters of tight junction genes in the nucleus, leading to the dissolution of tight junction. Our experiment also revealeda decrease in ZO-1 and occludin in the CS group according to the results of IHC and western blot, whereas CAY10603 treatment showed a significant improvement, indicating that CAY10603 can adjust TJs by regulating the expression of ZO-1 and occludin.

In the present study, HDAC6 expression in the lungs increased after CS exposure, which may be a response to enhanced protein ubiquitination and acetylation. The expression of NRF2 [56] and SIRT1 [57] is decreased in the lungs of patients with COPD. Lam et al. [19]conducted a study in which Nrf2^–/–^ mice exhibited elevated baseline HDAC6 expression in the lungs that was further augmented upon exposure to CS, while increased basal and CS-induced protein ubiquitination was observed in the lungs of Nrf2^–/–^ mice. Additionally, the acetylation of HDAC6 was increased in lung homogenates from Sirt1^+/–^ mice, suggesting that SIRT1 functions as a deacetylase of HDAC6. Exposure of Sirt1^+/–^ mice to CS also resulted in increased expression of HDAC6 in lung homogenates. Collectively, these findings suggest a relationship between the regulation of HDAC6 expression and the acetylation state, as well as the clearance of ubiquitinated proteins in the lung.

As a member of the histone deacetylases, HDAC6 functions as a deacetylase that mainly targets non-histone proteins in the cytoplasm and non-enzymatic functions regulated by the ubiquitin-proteasome system [58]. Various substrates of HDAC6 have been found, including α -tubulin, cortactin, Hsp90, β -catenin, RIG-I, Ku70, HSPA5, HMGN2, PrxI, and Tat [30]. HDAC6 is associated with the occurrence and development of a variety of diseases, including neurodegenerative diseases [59], cancer [60], cardiovascular diseases [61], renal fibrosis [49], cystogenesis [62], and inflammation [63]. Studies on respiratory diseases have mainly focused on lung cancer, whereas there have been few studies related to COPD. Wang et al. [64]. found that HDAC6 deacetylates the epidermal growth factor receptor (EGFR) and plays an important role in the control of cell proliferation in lung adenocarcinoma. Moreover, a study by Deskin et al. [65]. found that HDAC6 regulated EMT in non-small cell lung cancer (NSCLC) by the mediating TGF-β-Notch signaling cascade. These signaling pathways also regulate EMT in COPD; therefore, we explored the role of HDAC6 in regulating EMT in COPD. Lam et al. [19]. reported that HDAC6 is an important regulator of autophagy-mediated ciliary shortening during CS exposure. Su et al. [66] suggested that HDAC6 upregulated collagen synthesis and the proliferation of bronchial smooth muscle cells (BSMCs), leading to airway remodelling in patients with COPD. In this study, we found that CAY10603 treatment reduced the expression of E-cadherin and increased the expression of α-SMA induced by TGF-β1 in HBE cells, which was mediated via the suppression of Smad2 and Smad3 phosphorylation. CAY10603 attenuated TGF-β1-induced EMT in HEB cells and is expected to be a potential treatment for COPD in the future.

However, this study has some limitations that warrant discussion. For instance, we did not fully clarify how HDAC6 acts on tight junction proteins. Whether HDAC6 regulates EMT in airway epithelial cells through other mechanisms, such as adherens junctions, will be the focus of our subsequent research.

## Conclusion

In conclusion, our results revealed that the HDAC6-selective inhibitor CAY10603 inhibited CS-induced small airway remodelling by regulating epithelial barrier dysfunction and reversing EMT via the TGF-β1/Smad2/3 signalling pathway and CAY10603 treatment could significantly protect against CS-induced airway remodeling and emphysema.

### Electronic supplementary material

Below is the link to the electronic supplementary material.

Supplementary Material 1: Supplementary original blot images.

## Data Availability

The data used to support the findings of this study are available from the corresponding.

## Abbreviations

COPD: Chronic obstructive pulmonary disease
EMT: Epithelial-to-mesenchymal transition
HDAC: Histone deacetylase
CS: Cigarette smoking
CON: Control group
BAL: Bronchoalveolar lavage
H&E: Hematoxylin and eosin
PAS: Periodic acid–Schiff
MLI: Mean linear intercept
MAN: Mean alveolar number
Pbm: Perimeter of bronchial basement membrane
CSE: Cigarette smoke extract
HBE: Human lung bronchial epithelial cell line
FBS: Fetal bovine serum
ANOVA: Analysis of variance
EndMT: Endothelial to mesenchymal transition
ROS: Reactive oxygen species
Hh: Hedgehog
uPAR: Urokinase plasminogen activator receptor
TJs: Tight junctions
AJs: Adherens junctions
JAMs: Junction adhesion molecules
ZO: Zonula occludens
EGFR: Epidermal growth factor receptor
NSCLC: Non-small cell lung cancer
BSMCs: Bronchial smooth muscle cells
