# Supplementary figures and images for: HDAC6-selective inhibitor CAY10603 ameliorates cigarette smoke-induced small airway remodeling by regulating epithelial barrier dysfunction and reversing
Source: Respir Res. 2024 Feb 5;25:66. doi: 10.1186/s12931-024-02688-3 (PMC10840206; doi:10.1186/s12931-024-02688-3)

**Original Western blots**

**Figure. 1A**

**
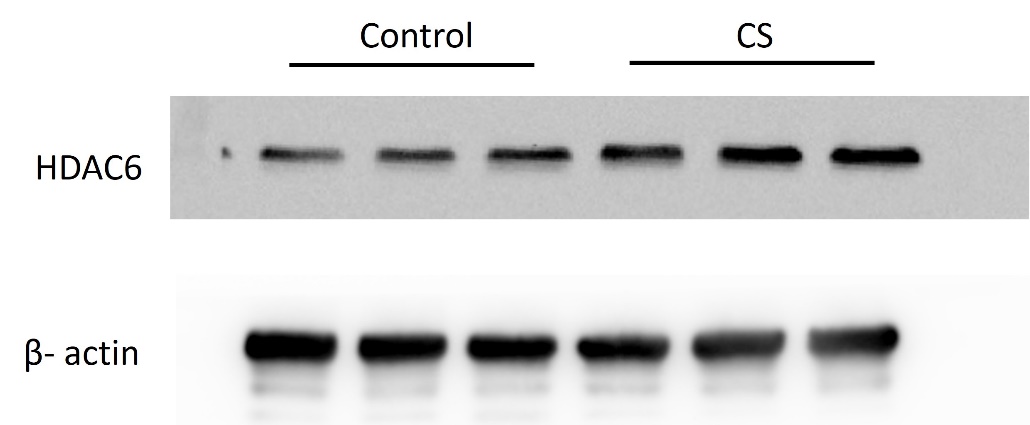
**

**Figure. 2B**

**
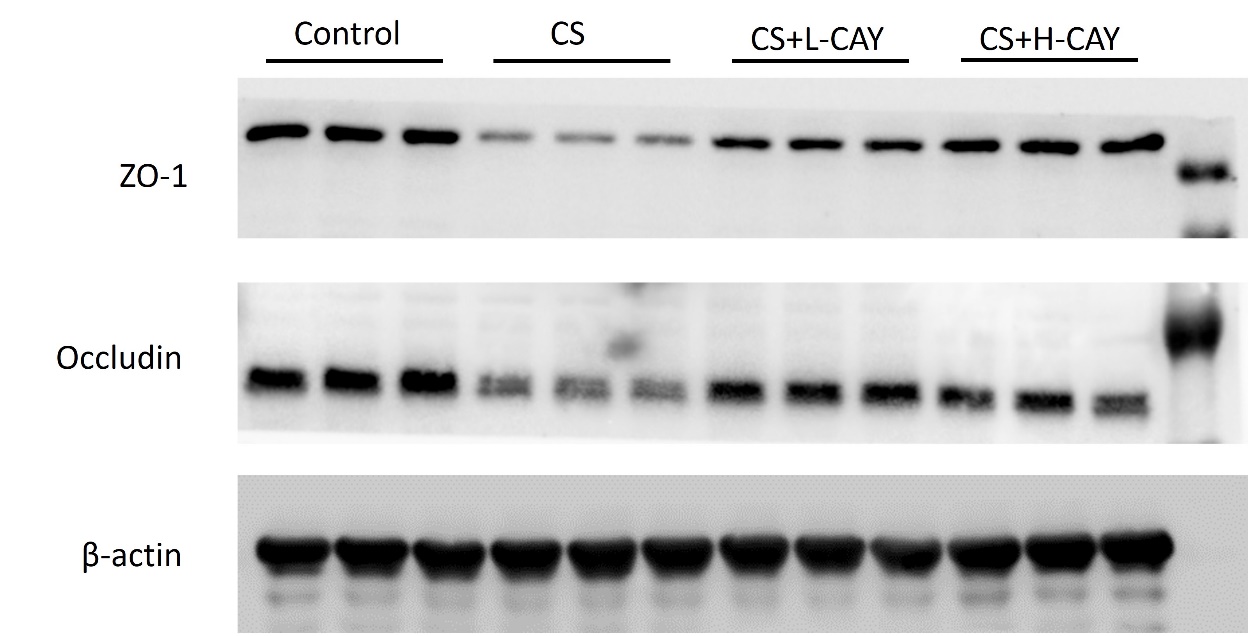
**

**Figure. 6b**

**
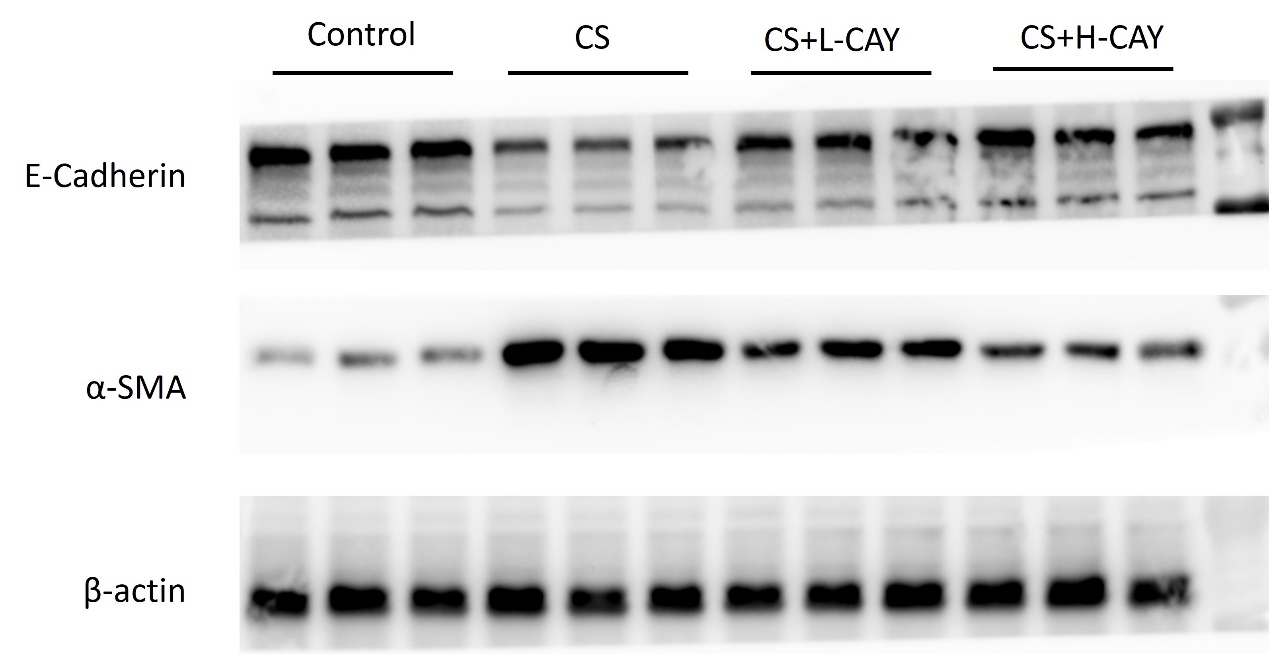
**

**Figure. 8A**

**
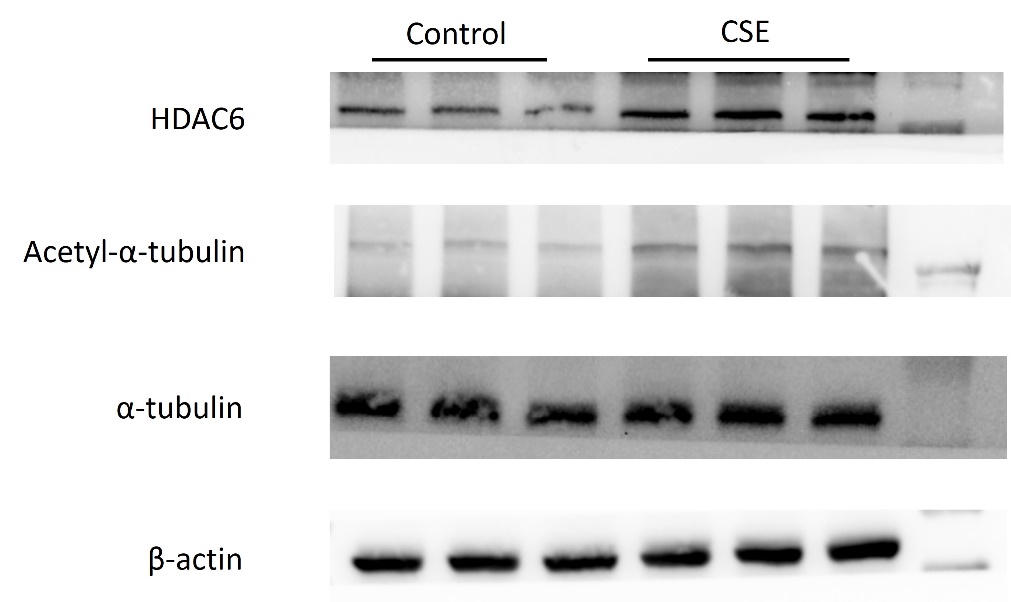
**

**Figure. 8E**

**
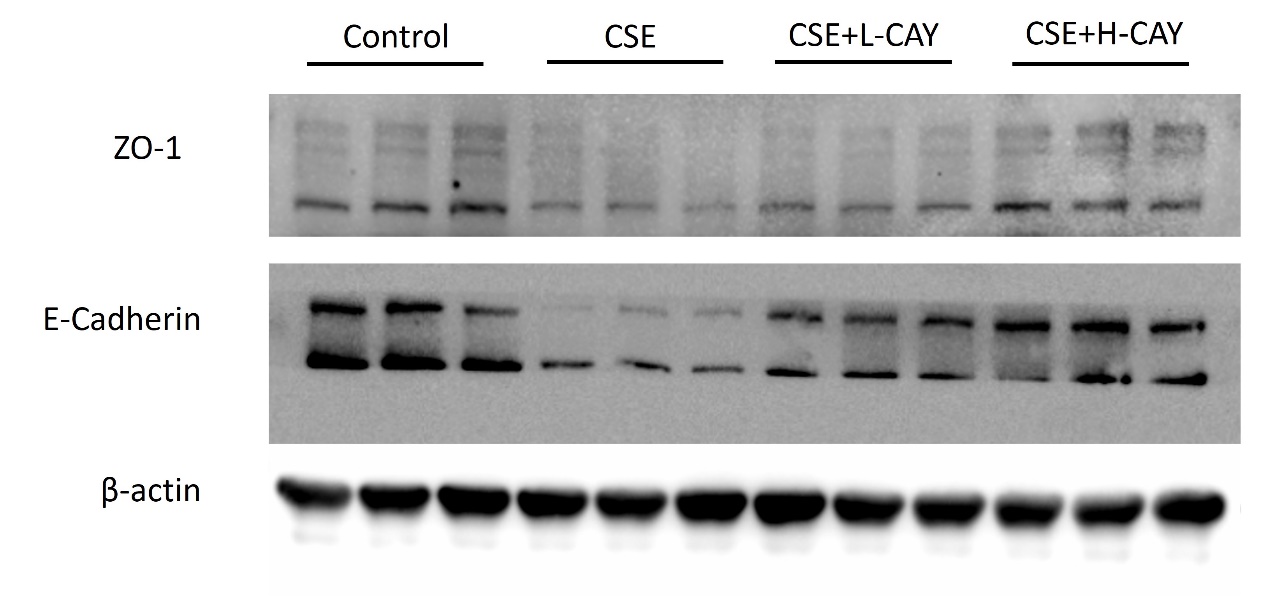
**

**Figure. 8F**

**
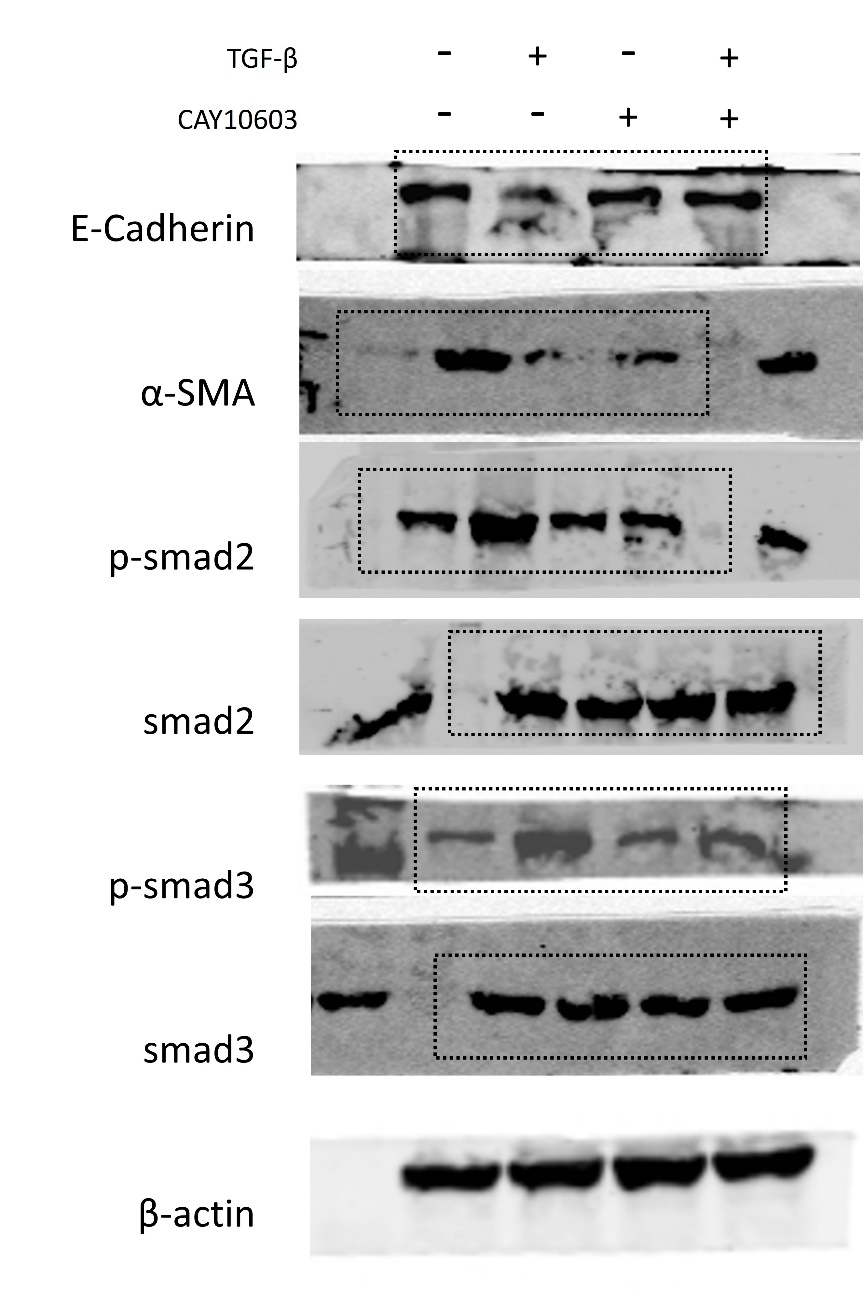
**

Supplement: Supplementary file 1 — Supplementary Material 1: Supplementary original blot images. [file 12931_2024_2688_MOESM1_ESM.docx]
